# A longitudinal multi-scanner multimodal human neuroimaging dataset

**DOI:** 10.1038/s41597-022-01386-3

**Published:** 2022-06-14

**Authors:** Colin Hawco, Erin W. Dickie, Gabrielle Herman, Jessica A. Turner, Miklos Argyelan, Anil K. Malhotra, Robert W. Buchanan, Aristotle N. Voineskos

**Affiliations:** 1 Campbell Family Mental Health Institute, Centre for Addiction and Mental Health, Toronto, ON Canada; 2 Department of Psychiatry, University of Toronto, Toronto, ON Canada; 3 Department of Psychology & Neuroscience Institute, Georgia State University, Atlanta, GA United States; 4 The Zucker School of Medicine at Hofstra/Northwell, Hempstead, NY United States; 5 Maryland Psychiatric Research Center, University of Maryland School of Medicine, Baltimore, MD United States

**Keywords:** Cognitive neuroscience, Computational neuroscience, Magnetic resonance imaging

## Abstract

Human neuroimaging has led to an overwhelming amount of research into brain function in healthy and clinical populations. However, a better appreciation of the limitations of small sample studies has led to an increased number of multi-site, multi-scanner protocols to understand human brain function. As part of a multi-site project examining social cognition in schizophrenia, a group of “travelling human phantoms” had structural T1, diffusion, and resting-state functional MRIs obtained annually at each of three sites. Scan protocols were carefully harmonized across sites prior to the study. Due to scanner upgrades at each site (all sites acquired PRISMA MRIs during the study) and one participant being replaced, the end result was 30 MRI scans across 4 people, 6 MRIs, and 4 years. This dataset includes multiple neuroimaging modalities and repeated scans across six MRIs. It can be used to evaluate differences across scanners, consistency of pipeline outputs, or test multi-scanner harmonization approaches.

## Abstract (summary)

**Table:** 

Measurement(s)	Three-Dimensional Spoiled Gradient MRI • Functional Magnetic Resonance Imaging • Diffusion Weighted Imaging
Technology Type(s)	T1 MRI • fMRI • Diffusion MRI
Sample Characteristic - Organism	homo sapiens
Sample Characteristic - Location	Canada and United States

## Background & Summary

Multi-site study designs are highly beneficial in combining resources across centers to maximize research participant recruitment. Such approaches have proven powerful for acquiring the large samples needed for transformative research. Multi-site collaboration is vital in the context of techniques such as neuroimaging, where data acquisition is both expensive and time-consuming, and in psychiatric research, where the available patient population at any given center is limited. However, multi-site neuroimaging research also suffers from some inherent challenges. Differences in signal characteristics across scanners, including scanners of the same manufacturer and model, can pose a challenge when combining data collected across multiple sites^1,2^. Furthermore, even within a single-site study, scanner upgrades and related changes can have impacts on data^3^; this problem is magnified when several sites are contributing data and potentially undergoing upgrades during the course of a study. Evaluating approaches to harmonize data across sites is essential as an increasing number of neuroimaging datasets include data collected across different scanners. One tool for validating harmonization approaches is to examine data from the same individuals collected across multiple scanners.

Another issue that has been rising to the forefront in neuroimaging research is low replicability^4–6^. Substantial efforts have been devoted to using neuroimaging approaches to develop ‘biomarkers’ of illness^7–10^. This has led to a proliferation of large scale, multi site neuroimaging studies, such as the Alzheimer’s Disease Neuroimaging Initiative (ADNI)^11^, the Canadian Dementia Imaging Protocol (CDIP)^12^, and the UK Biobank^13^. However, any such neuroimaging biomarkers are only clinically useful if they are stable/reliable measures. The stability of neuroimaging measures can be assessed via scan-rescan approaches. While some structural MRI measures such as cortical thickness seem to have high stability^14–16^, functional measures, such as task activity or functional connectivity, typically show low reliability when repeated in the same individual^14,17^. Measures such as functional connectivity between two specific brain regions are statistical values of inherently noisy signals; it may not be expected that such measures would show high stability. But despite poor scan-rescan reliability, functional connectivity patterns are individually identifying^14,18^. This demonstrates that useful signal is present amongst the noise, and within-subject scan-rescan variability may be lower than between-subject variability. Establishing useful neuroimaging biomarkers requires establishing the stability/reliability of such measures, as well as accounting for non-biological factors such as different signal characteristics across scanners. This is especially critical given the growth of large sample multi-site neuroimaging datasets.

We have been exploring biomarkers of social cognitive dysfunction in schizophrenia through the NIMH RDoC study ‘Social Processes Initiative in the Neurobiology of the Schizophrenias’ (SPINS)^19–21^. The SPINS study sample included neuroimaging data collected across three sites to maximize the available number of participants. To examine signal differences across MRIs, three “human phantoms” travelled to each of the three sites and underwent the imaging protocol. This process was repeated on an annual basis, during which one new “human phantom” was added and three new (PRISMA) scanners were introduced. We previously used a subset of this data to assess neuroimaging metrics across individuals and scanners^14^. Using a clustering approach, we noted that, with minimal correction for different scanners, an individual’s repeated scans are reliably clustered together. However, essential sources of MRI scanner variability were also present; particularly, the PRISMA scanners had substantial differences in diffusion metrics compared to the non-PRISMA scanners.

In total, this dataset consists of 30 scanning sessions across four individuals on six MRIs across four years. It provides a valuable resource for considering the MRI scanner’s effects on functional and structural neuroimaging metrics. While other datasets are available with larger numbers of participants or a greater number of scans^17,22^, this dataset has a relatively unique combination of the inclusion of functional, structural, and diffusion data collected across several MRI models over time. This provides an opportunity to examine site effects in a more naturalistic way, in a dataset that emerged from a multi-site study rather than being systematically designed and controlled for site effects. In this way, this dataset represents a ‘real world’ example of the challenges of integrating data across sites.

## Methods

### Data collection

MRI scans were collected annually across three participating sites starting in 2014 and ending in 2018. Participants traveled to each site and performed an MRI scan. At all three sites, Siemens Prisma MRIs were made available part way through the study. As a result, data from six MRIs is included in the dataset; each site changed MRI scanners mid-study. Each MRI is identified via a three-letter identifier in the file names (see below and Table 1). The three study sites were the Center for Addiction and Mental Health, affiliated with the University of Toronto, the Maryland Psychiatric Research Center (MPRC) affiliated with the University of Maryland School of Medicine, and Zucker Hillside Hospital, affiliated with the Zucker School of Medicine at Hofstra/Northwell. The original MRI at the Center for Addiction and Mental Health was a General Electric 750w Discovery 3 T, referred to as CMH; a Siemens Prisma (referred to as CMP) was made available in year four at the Toronto Neuroimaging Facility (ToNI) of the University of Toronto, Department of Psychology. The original MRI model at the Maryland Psychiatric Research Center was a Siemens Tim Trio 3 T (referred to as MRC); they later upgraded to a Siemens Prisma (referred to as MRP) for year three. Zucker Hillside had a General Electric 750 Signa 3 T (refer to a ZHH) at study start and upgraded to a Siemens Prisma (referred to as ZHP) for year three. Scans were performed annually, though in 2018 (year 4) scans were only performed on CMP (to collect data from the newly available Prisma scanner). Scan sites, scanner model and years at which scans were performed on each MRI are presented in Table 1.

**Table 1:** Scanners by site.

Site	MRI Scanner	Years	Scanner ID
Toronto	General Electric 750w Discovery 3 T	1,2,3	CMH
Toronto	Siemens Prisma 3 T	4	CMP
Maryland	Siemens Tim Trio 3 T	1,2	MRC
Maryland	Siemens Prisma 3 T	3	MRP
New York	General Electric 750 Signa 3 T	1,2	ZHH
New York	Siemens Prisma 3 T	3	ZHP

### Participants

**Fig. 1:**
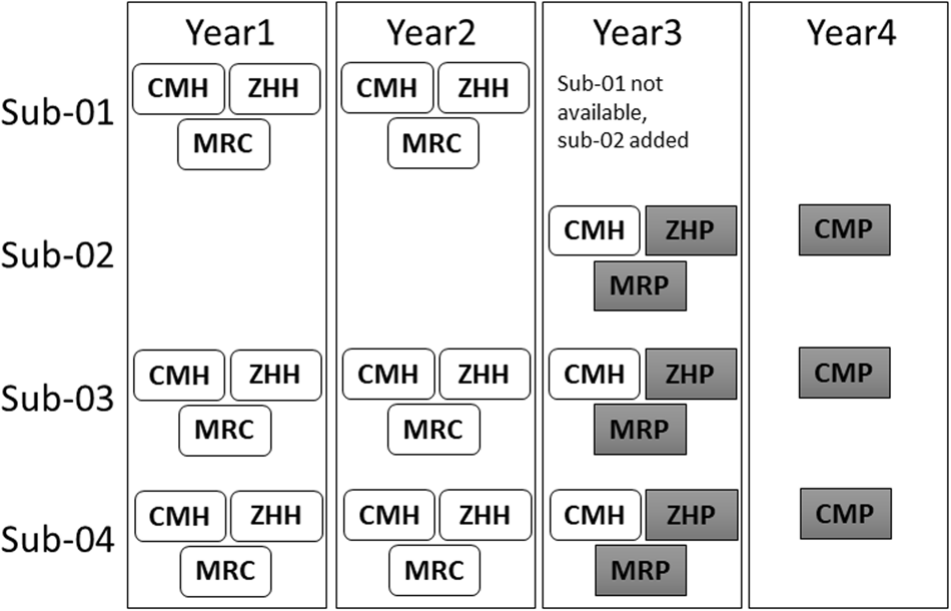
Schematic of participants scanned at each MRI by year. Prisma scanners are shown in gray.

Data are available from four healthy male participants, aged 34 to 59 during study start (aged 38 to 63 at study end). Participants had no history of psychiatric, neurological, or major chronic illness. Participants sub-01, sub-02, and sub-03 were collected at year one (study start) and during year two on scanners CMH, MRC, and ZHH. However, sub-03 was not available in year three and was thus replaced with sub-04. In year three, sub-01, sub-02, and sub-04 were scanned on CMH, MRP, and ZHP. In year four, participants sub-01, sub-02, and sub-04 were scanned at CMP only to provide data on an additional PRISMA scanner. A total of 30 MRI sessions were completed across the study. A schematic of the participant’s scanner schedule by year is presented in Fig. 1. All participants signed informed consent, including explicit consent to share data on a public repository, and the study had IRB approval at all sites.

### Prospective data harmonization

Data were prospectively harmonized to the limits of available hardware at each site by matching scan parameters. When exact parameters were unable to be matched due to hardware capabilities or limitations, the closest appropriate value was selected. As the Siemens Prisma scanners were identical models, it was possible to perfectly match scan parameters across sites.

### MRI scans

Each participant underwent MRI scans consisting of a T1-weighted (T1w) anatomical MRI, a diffusion-weighted MRI, and a 7-minute resting state functional MRI. The parameters for all sequences across all scanners were matched as closely as possible given the available hardware and specifics of each scanner. T1w anatomical scans used the manufacturer-specific fast gradient echo sequences (Siemens MRIs MRC, MRP, ZHP, and CMP: MPRAGE; GE MRIs CMH, ZHH: BRAVO). T1w scan parameters were TR = 2300 ms, 0.9 mm isotropic voxel size with no gap, interleaved ascending acquisition order, with TE from 2.78–3 ms, as determined by the scanner-specific hardware). As is standard practice to increase scan SNR at the MPRC site (Kochunov *et al*., 2006), three T1w scans were acquired and subsequently averaged into a single image prior to any preprocessing for the MRC and MRP scanners.

Diffusion weighted scans used an axial EPI dual spin echo sequence, with 60 gradient directions, b = 1000, five baseline scans with b = 0 (or six in the case of the PRISMA scanners), TR = 8800 ms, with the exception of ZHH where TR = 17700 ms; TE = 85 ms; FOV = 256 mm; in-plane matrix size was 128 × 128, 2.0 mm isotropic voxels with no gap). Resting fMRI scans lasted 7 minutes, with participants instructed to remain awake, eyes closed, and let their mind wander. The resting state fMRI was an EPI sequence of 212 volumes, TR = 2000 ms, TE = 30.0ms, FOV = 20 cm, 64 × 64 acquisition matrix, 40 slices of 4 mm thickness, interleaved ascending acquisition order. Relevant scan parameters are included in json files provided with each data record, and are presented in detail in Supplementary Table 1.

### BIDS derivatives (BIDS details)

All data are organized according to the brain imaging data structure (BIDS) formatting^23^. BIDS provides a standardized naming specification and folder structure to allow for easy reference and standardized pipelines. In the BIDs specification, a folder for each participant exists at the top level of the folder hierarchy, with scans/sessions embedded within. Each session folder then has a sub-folder for each scan type acquired.

Following data acquisition, DICOM files were exported from each site to a common XNAT repository, and visual QC was performed. DICOM images were then converted to nifti format via dcm2niix v1.0.20190410, and renamed/organized according to the current BIDS standards. The integrity of the BIDS formatting was then checked via the BIDS validator (https://bids-standard.github.io/bids-validator/). To preserve participant anonymity, T1 scans were defaced using pydeface 2.0.0 (https://github.com/poldracklab/pydeface).

## Data Records

Raw data has been uploaded to https://openneuro.org, in a repository entitled “Social Processes Initiative in Neurobiology of the Schizophrenia(s) Traveling Human Phantoms”^24^, accession number **ds003011**. The full link is https://openneuro.org/datasets/ds003011.

Following BIDS, data from each participant is stored within a separate folder, labeled by subject ID (sub-01, sub-02, sub-03, and sub-04). Within participant folders, separate folders exist for each session which that participant completed. Session folders are labeled in the form ses-[Year][Scanner], with year indicated by Y1, Y2, Y3, or Y4 (for years 1–4 respectively) and scanner as indicated on Table 2. Each session folder in turn includes folders for anat (T1), func (resting state fMRI), and dwi (diffusion MRI scan). Within each of these folders exists a nifti (.nii.gz) file for the image, and a.json file containing scan parameters (generated by dcm2niix). For the dwi folder, bvec and bval files are also included, containing the gradient directions and diffusion weighting respectively.

**Table 2:** Demographic details for each scan included in the dataset.

			Record	Participant	Scanner	Date	Age
sub-01	Y1	CMH	sub-01_ses-Y1CMH	sub-01	CMH	Fall 2014	52
sub-01	Y1	MRC	sub-01_ses-Y1MRC	sub-01	MRC	Fall 2014	52
sub-01	Y1	ZHH	sub-01_ses-Y1ZHH	sub-01	ZHH	Fall 2014	52
sub-01	Y2	CMH	sub-01_ses-Y2CMH	sub-01	CMH	Fall 2015	53
sub-01	Y2	MRC	sub-01_ses-Y2MRC	sub-01	MRC	Fall 2015	53
sub-01	Y2	ZHH	sub-01_ses-Y2ZHH	sub-01	ZHH	Fall 2015	53
sub-02	Y3	CMH	sub-02_ses-Y3CMH	sub-02	CMH	Fall 2016	39
sub-02	Y3	MRP	sub-02_ses-Y3MRP	sub-02	MRP	Fall 2016	39
sub-02	Y3	ZHP	sub-02_ses-Y3ZHP	sub-02	ZHP	Fall 2016	39
sub-02	Y4	CMP	sub-02_ses-Y4CMP	sub-02	CMP	Fall 2017	40
sub-03	Y1	CMH	sub-03_ses-Y1CMH	sub-03	CMH	Fall 2014	36
sub-03	Y1	MRC	sub-03_ses-Y1MRC	sub-03	MRC	Fall 2014	36
sub-03	Y1	ZHH	sub-03_ses-Y1ZHH	sub-03	ZHH	Fall 2014	36
sub-03	Y2	CMH	sub-03_ses-Y2CMH	sub-03	CMH	Fall 2015	37
sub-03	Y2	MRC	sub-03_ses-Y2MRC	sub-03	MRC	Fall 2015	37
sub-03	Y2	ZHH	sub-03_ses-Y2ZHH	sub-03	ZHH	Fall 2015	37
sub-03	Y3	CMH	sub-03_ses-Y3CMH	sub-03	CMH	Fall 2016	38
sub-03	Y3	MRP	sub-03_ses-Y3MRP	sub-03	MRP	Fall 2016	38
sub-03	Y3	ZHP	sub-03_ses-Y3ZHP	sub-03	ZHP	Fall 2016	38
sub-03	Y4	CMP	sub-03_ses-Y4CMP	sub-03	CMP	Fall 2017	39
sub-04	Y1	CMH	sub-04_ses-Y1CMH	sub-04	CMH	Fall 2014	59
sub-04	Y1	MRC	sub-04_ses-Y1MRC	sub-04	MRC	Fall 2014	59
sub-04	Y1	ZHH	sub-04_ses-Y1ZHH	sub-04	ZHH	Fall 2014	59
sub-04	Y2	CMH	sub-04_ses-Y2CMH	sub-04	CMH	Fall 2015	60
sub-04	Y2	MRC	sub-04_ses-Y2MRC	sub-04	MRC	Fall 2015	60
sub-04	Y2	ZHH	sub-04_ses-Y2ZHH	sub-04	ZHH	Fall 2015	60
sub-04	Y3	CMH	sub-04_ses-Y3CMH	sub-04	CMH	Fall 2016	61
sub-04	Y3	MRP	sub-04_ses-Y3MRP	sub-04	MRP	Fall 2016	61
sub-04	Y3	ZHP	sub-04_ses-Y3ZHP	sub-04	ZHP	Fall 2016	61
sub-04	Y4	CMP	sub-04_ses-Y4CMP	sub-04	CMP	Fall 2017	62
**sub-03**	**Y4**	**CMP**					

## Technical Validation

**Fig. 2:**
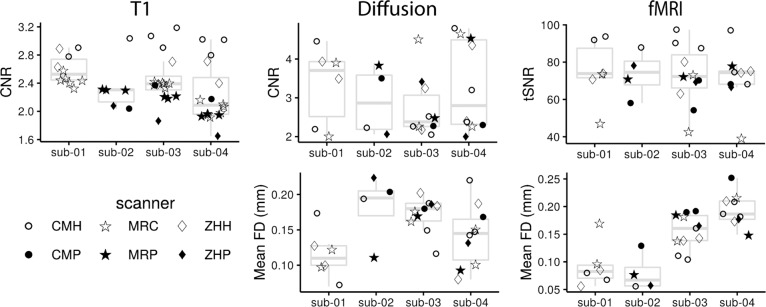
MRI quality metrics for each scan, organized by participant (boxplot). The scanner is identified via different markers. The bottom and top of the boxes correspond, respectively, to the first and third quartiles. The upper and lower whiskers extend to the largest/smallest values at a maximum of 1.5*IQR away from the box, and data beyond the ends of whiskers are outliers.

After MRI images were acquired, the scans were reviewed visually for artefacts and to ensure the diffusion scan directions were correct. To extract standardized image quality metrics (IQMs), the T1 and fMRI images were run through mriqc (version 0.14.2)^25^. IQMs were extracted from diffusion scans using FSL EDDY QUAD (Quality Assessment for DMRI)^26^. Data quality metrics are shown in Fig. 2. CNR (contrast-to-noise ratio) is an extension of signal to noise ratio (SNR), which evaluates the separation between gray and white matter tissue distributions. SNR was not used as a metric in this case as the bias field within the PRISMA scans distorts the SNR calculation. Mean framewise displacement (FD) is reported for DWI and fMRI. FD reflects head motion from frame-to-frame in an image that is acquired across time (i.e., fMRI or DWI). We report the mean of this framewise displacement for each scan. tSNR (time signal to noise ratio) is an extension of SNR over time. It is calculated as the bold signal across time divided by the temporal standard deviation.

Of the 30 scans included in this data set, 27 were part of a previously published paper examining differences between scanner and participants^14^. In that analysis, we performed hierarchical clustering on cortical thickness derived from the T1, basic diffusion metrics (white matter skeleton fractional anisotropy, FA), and resting state connectivity. In an initial clustering solution, diffusion data showed scanner specific clustering, with the PRISMA scan separating into a distinct cluster. However, when a simple correction for scanner was incorporated, all three modalities clustered by participant but not scanner, with the exception of two high motion resting state scans. This demonstrates that the data from all three scans was of sufficient quality to distinguish scans from different participants scanned repeatedly.

## Usage Notes

Given ongoing concerns over reliability and sample size in neuroimaging, multi-site studies have become a critical tool for advancing research. This can be especially true in research on clinical populations, which has resulted in tremendous growth of multi-site neuroimaging samples^11–13,20,27^, as well as in the identification of treatment relevant biomarkers or biotypes^28,29^. While the protocol used in the current dataset uses older imaging sequences, which are not in alignment with state of the art public data sets, such as human connectome or adolescence brain cognitive development^27,30^, clinical datasets, such as ADNI, more commonly have scanning parameters closer to those in the current protocol^11,12^. Large scale projects such as the ENIGMA consortium have demonstrated the power of building large samples from combining previously collected data^31,32^, often featuring older sequences such as those in the current data.One challenge of working on neuroimaging data collected across multiple sites can be differences in signal across MRIs, even from the same vendor or the same model^3,14,15,33–35^. For example, within this data set our previous work noted a large difference in diffusion metrics in the Prisma as compared to non-Prisma scanners^14^. Repeated scanning data sets, such as the one presented here, can be useful to validate approaches intended to minimize MRI specific signal differences while retaining individually unique variance. While there has been a growing number of scan-rescan data sets at a single site^36^ or incorporating data from multiple sites but rescanning on the same MRI^37^, there remains a relative paucity of openly available multi-site travelling human datasets^17,38,39^. This data adds an additional openly available resource for evaluating the effects of different scanners within the same individuals, including the combination of structural, functional, and diffusion metrics which are not generally present in a single travelling human sample.

Repeated multi-site data sets also have the potential to address issues around stability and reliability of neuroimaging metrics^1,5,16,17^. Whether considering clinical populations of typically developing individuals, valid measurements are essential to understanding the human brain. These challenges can be exacerbated in clinical populations or when scanning older or younger populations, who often show greater motion^40,41^. Moreover, signal quality issues and higher motion related to behavior variability of interest, such as impulsivity or other clinical scales, can influence the assessment of brain-behavior relationships^42,43^. However, despite challenges in reliability of measurements, especially in fMRI^44,45^, important and replicable individual variability can be observed within these data^18,22^. Repeat scanning data sets can be used to help evaluate analytical approaches designed to minimize noise signals (e.g. motion) or pull important individual sources of variability from different MRI signals. For example, analytical frameworks which decrease within-subject variability (while accounting for potential within-subject noise sources) may represent more valid measures for individually meaningful brain signals.

This data set adds another resource to a growing list of publicly available rescan data sets. The novel features of this data set include the multi-modal data available and the fact that scans were collected across several years, MRIs, and across scanner upgrades (a common issue during longitudinal studies and long-term data collection).

## Supplementary information

Supplementary Table 1

## Data Availability

All tools used are open source and available at their respective references. The code for generating plots of quality control data (and the respective source data) can be found at https://github.com/TIGRLab/human_phantoms_plots.
